# Norditerpene Natural Products from Subterranean Fungi with Anti-Parasitic Activity

**DOI:** 10.3390/microorganisms13112527

**Published:** 2025-11-04

**Authors:** Alexandra Kolas, Yudi Rusman, Ana C. R. G. Maia, Jessica M. Williams, Jiashu Xie, Roshan Katekar, Fernanda G. Fumuso, Alexis Cotto-Rosario, Chidiebere N. Onoh, Hanen Baggar, Mary L. Piaskowski, Christian Baigorria, Raphaella Paes, Debopam Chakrabarti, Lyssa J. Weible, Kayode K. Ojo, Roberta M. O’Connor, Christine E. Salomon

**Affiliations:** 1 Department of Veterinary and Biomedical Sciences, University of Minnesota, St. Paul, MN 55108, USA; 2 Center for Drug Design, University of Minnesota, Minneapolis, MN 55455, USA; 3 Division of Molecular Microbiology, Burnett School of Biomedical Sciences, University of Central Florida, 12722 Research Parkway, Orlando, FL 32826, USA; 4 Center for Emerging and Reemerging Infectious Diseases (CERID), Division of Allergy and Infectious Diseases, Department of Medicine, University of Washington, Seattle, WA 98109, USA; 5 Department of Global Health, University of Washington, Seattle, WA 98195, USA

**Keywords:** *Cryptosporidium*, *Toxoplasma*, apicomplexa, anti-parasitic, natural product, norditerpene, oidiolactone

## Abstract

*Cryptosporidium* is a waterborne gastrointestinal parasite that causes diarrheal disease worldwide. Currently, there are no effective therapeutics to treat cryptosporidiosis. Since natural products are a known source of anti-parasitic compounds, we screened a library of extracts and pure compounds isolated from bacteria and fungi collected from subterranean environments for anti-*Cryptosporidium* activity. Seven norditerpene lactones isolated from the fungus *Oidiodendron truncatum* collected from the Soudan Iron mine in Minnesota showed potent activity and were further tested to identify the most active compounds. The availability of a diverse suite of natural structural analogs with varying activities allowed us to determine some structure–activity relationships for both anti-parasitic activity and cytotoxicity. The two most potent compounds, oidiolactones A and B, had EC_50_s against *C. parvum* of 530 and 240 nM, respectively, without cytotoxicity to host cells. Both compounds also inhibited the related parasite *Toxoplasma gondii*. Oidiolactone A was active against asexual, but not sexual, stages of *C. parvum*, and killed parasites within 8 h of treatment. This compound reduced *C. parvum* infection by 70% in IFNγ−/− mice, with no signs of toxicity. The high potency, low cytotoxicity, and in vivo activity combined with high production and synthetic accessibility make these oidiolactones attractive scaffolds for the development of new anti-*Cryptosporidium* therapeutics.

## 1. Introduction

*Cryptosporidium parvum* is a zoonotic, waterborne gastrointestinal parasite that causes significant morbidity and mortality in humans and livestock in both developing and industrialized countries [1,2]. The parasite is acquired by ingestion of food or water contaminated with oocysts, the environmentally resistant stage of the parasite [3]. *Cryptosporidium* replicates in intestinal epithelial cells, first undergoing asexual replication followed by sexual development [4] and production of oocysts that are subsequently released in the feces [3]. The infection is perpetuated by autoinfection with thin-walled oocysts; diarrhea caused by *Cryptosporidium* can last up to 2–3 weeks, resulting in significant morbidity, and sometimes mortality [5,6].

In the United States, *Cryptosporidium* was responsible for the largest outbreak of waterborne diarrheal disease [7], and worldwide, it remains a common cause of waterborne diarrheal illness [1,8]. Currently, the chance of exposure to the pathogen is high, even in industrialized countries [1,8], and continues to increase [9]. Immunocompromised patients are highly susceptible to *Cryptosporidium* infection, and unless the immune system can be reconstituted, the infection results in prolonged, chronic, and sometimes fatal diarrhea [9]. In developing countries, *Cryptosporidium* parasites are the second most common cause of diarrhea in infants and the only gastrointestinal pathogen associated with death in toddlers [10]. Currently, there is no effective therapeutic to treat these at-risk populations [9]. The only FDA-approved drug, nitazoxanide, is ineffective in immunocompromised patients [11], and clofazimine, a repurposed leprosy drug with activity against *Cryptosporidium*, failed in clinical trials in HIV-*Cryptosporidium* co-infected adults [12]. Critically, there are few anti-*Cryptosporidium* drugs in the pipeline [13]. Given the high failure rate of candidate therapeutics as they progress through pre-clinical and clinical development, more anti-*Cryptosporidium* compounds need to be discovered and investigated [14].

*Cryptosporidium parvum* is also a significant problem in veterinary medicine. The parasite is endemic in cattle operations worldwide [15,16] and responsible for ~40% of neonatal calf enteritis [17], causing a significant economic impact on farmers. Moreover, cattle and dairy farms serve as an environmental reservoir of this zoonotic pathogen; contamination of recreational waters by farm runoff has been documented [13]. Currently, there are no drugs available to treat cryptosporidiosis in neonatal ruminants [17]. Therefore, targeted development of effective therapeutics for cryptosporidiosis remains both a veterinary and medical imperative.

Throughout history, natural products have played a pivotal role in the discovery and development of treatments for parasitic disease. Plants, mushrooms, and plant-based remedies have been used by traditional healers as essential medicines to treat infectious diseases [18]. The discovery and development of quinine from the bark of cinchona “fever trees” [19] and artemisinin from sweet wormwood (*Artemesia annua*) [20] revolutionized treatments for malaria across the globe. The more recent example of the discovery of the avermectins from *Streptomyces avermitilis* [21] and subsequent development into ivermectin for the treatment of nematode infections highlights the potential of microbial sources of bioactive lead compounds for drug development [22]. The rich diversity of both chemical structures and source organisms has proven to be a valuable resource for the discovery of potent anti-parasitic compounds with novel mechanisms of action [23,24].

As part of a program to identify new natural product inhibitors of *C. parvum*, we screened a library of 192 crude extracts, enriched fractions, and pure compounds from fungi and bacteria collected from a subterranean iron mine in Tower, Minnesota. The source microbial strains were selected for this pilot screen due to their anti-fungal activity identified in a previous biocontrol study [25]. We found several related norditerpene compounds possessing anti-*C. parvum* activity; two of these compounds exhibited submicromolar activity against this parasite and the related apicomplexan, *Toxoplasma gondii*. Atypically for most natural products, these two active oidiolactones are produced in high titers by the *Oidiodendron truncatum* fungus (>60 mg/Kg) and are the most abundant secondary metabolites produced in solid culture by this strain under our conditions. The plentiful availability of these compounds and potent activities encouraged us to further assess their potential via measurement of pharmacokinetic parameters and additional characterization of their anti-parasitic activity in vitro and in vivo. In addition to testing with *C. parvum* and *T. gondii*, we included the malarial parasite *Plasmodium falciparum* to evaluate the specificity of activity across diverse Apicomplexan parasites. We also included *Giardia lamblia*, a more distantly related extracellular intestinal parasite to further assess specificity. Comparing activities across a range of related parasites provides a framework for identifying potential conserved molecular targets and provides information about the application and limitations of these compounds as potential lead pharmacophores for developing new anti-parasitic drugs.

## 2. Materials and Methods

**Compounds and extracts:** The isolation and structural elucidation of compounds **1–9** and **11–14** were previously described [25]. An additional analog, yukonin (**10**), was also included in bioactivity screening. The fungus *O. truncatum* (MN0802960) was isolated from the 25th level of the Soudan Iron Mine in Tower, Minnesota and cultured on potato dextrose agar for ten days. Approximately 1/8th of a fully colonized agar plate was chopped into small pieces and vortexed in 5 mL of PBS in a 50 mL conical tube and used to inoculate 200 g of rice media (50% rice/water by weight) in a 1 L flask, which was incubated for 30 days at room temperature (~22 °C). Methanol and ethyl acetate extracts (3 × 300 mL each) of the cultures were combined, dried, and successively partitioned with ethyl acetate, n-hexanes, and butanol. The ethyl acetate soluble fraction was separated using medium pressure flash chromatography (Teledyne ISCO Combiflash^®^ Rf, Lincoln, NE, USA; Solid phase Redisep^®^ Rf 24 g silica, Lincoln, NE, USA; gradient elution from 0 to 100% of EtOAc/n-Hexane for 35 min; flow rate of 25 mL/minute). Fractions were pooled into 18 fractions (**F1**–**F18**) based on TLC analysis. Fraction **F10** (260.4 mg) was further separated using semi-preparative HPLC (gradient elution from 25 to 60% acetonitrile/H_2_O for 23 min; flow rate of 3 mL/minute, column: Vision HT-C18 classic, 10 × 250 mm) to generate **F.10.11.** (12.2 mg). Fraction F.10.11 was then subjected to another HPLC separation (gradient elution from 35 to 57.5% acetonitrile/H_2_O for 9 min; flow rate of 1 mL/minute, column: Altima HP-C18, 4.5 × 250 mm) to yield yukonin (**10**, 1.25 mg), which was identified by comparison of NMR and mass spectrometry data to the literature values [26]. 

**Parasites and host cells:** Iowa strain transgenic *C. parvum* oocysts expressing nanoluciferase (Cp-NLuc) were acquired from the University of Arizona, Tuscon, AZ (https://acbs.arizona.edu/cryptosporidium-production-laboratory). Oocysts were used within 3 months of acquisition. Before use, the oocysts were bleached and washed as previously described [27]. 

Human ileocecal colorectal adenocarcinoma cells (HCT-8, ATCC® CCL244™, Manassis, VA, USA) were maintained as recommended and subcultured no more than 30 times for *C. parvum* experiments. The type I RH strain of *T. gondii*, expressing green fluorescent protein and luciferase (TgRH-Luc:GFP), was obtained from Jeroen Saeij at the University of California, Davis, CA, USA. TgRH-Luc:GFP parasites were maintained in human foreskin fibroblasts (ATCC SCRC-1041^TM^) as described in [28]. *Plasmodium falciparum* cultures were maintained following a modified Trager and Jensen protocol [29,30]. The multi-drug-resistant *P. falciparum* line Dd2 was grown in RPMI 1640 supplemented with 25 mM HEPES pH 7.4, 26 mM NaHCO_3_, 2% dextrose, 15 mg/L hypoxanthine, 25 mg/L gentamicin, and 0.5% Albumax II (Fisher Scientific, Waltham, MA, USA) in human A+ erythrocytes. Cultures were maintained at 4% hematocrit at 37 °C with 5% CO_2_. Axenic culture of wild-type *G*. *lamblia* WBC6 (ATCC 50803) and *G*. *lamblia* CBG99 luciferase-based reporter strains were used in this study [31,32].

**Antiparasitic and cytotoxicity assays:** All compounds were initially tested at a single concentration against *C. parvum* and *T. gondii*, and those with >50% inhibition were further tested at multiple concentrations to determine the EC_50_ values. Oidiolactone A was additionally studied using immunofluorescence assays with both species, tested in excystation and invasion assays with *C. parvum*, and tested in vivo using an acute mouse model of *C. parvum* infection. To assess the specificity of antiparasitic activity, the compounds were also tested against *P. falciparum* and *G. lamblia*. Each parasite lab also tested the compounds against the relevant host mammalian cell lines for cytotoxicity, and the specific methodology for each is described below. The pharmacokinetics experiments were done with oidiolactones A and B to help inform the dosing schedule and predict the oral bioavailability for the in vivo inhibition studies.

***C. parvum* growth inhibition assays:** The in vitro growth inhibition assay of *C. parvum* has been previously described [33]. Briefly, clear-bottom, white-sided 96 well plates were seeded with HCT-8 cells. Once the cells reached between 50% and 75% confluency, they were infected with Cp-NLuc oocysts (10,000/well) in complete media containing 0.6% taurocholate. At 24 h post-infection (hpi), the infected cells were treated with compounds at various concentrations. DMSO was used as a vehicle control in parallel. Parasite growth was determined at 72 hpi by measuring relative luminescence units (RLUs) using Promega Nano-Glo substrate (Promega, Madison, WI, USA) and a SpectraMax^®^ L Microplate Reader (Molecular Devices, San Jose, CA, USA). The percent inhibition of parasite growth was calculated as [(RLU_DMSO_ − RLU_compound_)/RLU_DMSO_] × 100. 

To determine -static versus -cidal activity, the infection assay was conducted as described, but infected cells were treated with compounds for 30 min up to 24 h. The compounds were removed after the designated times, and infection was quantified at 72 h post-infection. To determine the activity of the compounds against asexual and sexual stages of the parasite, compounds were added for 4 h windows at times corresponding to asexual or sexual development. Parasite proliferation was then determined at 72 h post-infection. For all assays, each sample was run in triplicate, and three biological replicates were performed.

***T. gondii* growth inhibition assays:** The *T. gondii* inhibition assay was described previously [34]. Briefly, HFF cells were seeded onto white-sided, clear-bottom 96 well plates, grown to confluence, and then 5000 TgRH-Luc:GFP parasites were added to each well. At 24 hpi, compounds or DMSO were added. The growth of the parasites was determined by relative luminescence at 48 hpi using Promega Bright-Glo (Promega, Madison, WI, USA) substrate. Plates were read, and the percent inhibition of parasite growth was calculated as described above. Determination of -static versus -cidal activity was conducted as indicated above, with parasite growth quantified after 48 h of infection. Each sample was run in triplicate, and each experiment was repeated three times.

***Plasmodium falciparum* growth inhibition assay:** Antiplasmodial EC_50_ results were determined using a fluorescence-based SYBR Green I assay performed using asynchronous cultures [30,35]. For screening, parasites were diluted to 1% parasitemia and 2% hematocrit, then incubated with serial dilutions of compounds in microtiter plates for 72 h under standard growth conditions. Plates were subsequently frozen at −80 °C to facilitate lysis. After thawing, plates were incubated with 1× SYBR Green I in lysis buffer (20 mM Tris-HCl, 0.08% saponin, 5 mM EDTA, 0.8% Triton X-100) for 45 min at room temperature. Fluorescence was measured at an excitation wavelength of 485 nm and emission wavelength of 530 nm in a Synergy Neo2 multimode reader (BioTek, Winsooki, VT, USA). Relative fluorescence units (RFUs) were normalized based on chloroquine-treated and no treatment controls. Serial dilutions of compounds were prepared in RPMI with final assay conditions of ≤0.2% DMSO. Each assay was conducted in triplicate with three biological replicates. Z′-factors in these assays were >0.7. 

**Compound inhibition assay against *Giardia lamblia* WBC6 and CBG99 trophozoites:** Biological replicates of growth inhibition assays were performed using wild-type *G*. *lamblia* WBC6 and *G*. *lamblia* CBG99 trophozoites at 37 °C under reduced oxygen conditions in a sealed 0.5 mL airtight sterile tube (USA Scientific, Ocala, FL, USA), following the susceptibility assay described below. *G*. *lamblia* trophozoites were grown in TYI-S-33 medium supplemented with 10% bovine serum and 0.05 mg/mL bovine bile, as previously described [31,36]. A 3-fold serial concentration of pure compounds starting at 29 µM was screened against 75,000 cells/mL of parasites in modified TYI-S-33 medium with a final dimethyl sulfoxide (DMSO) concentration of 0.14%. Metronidazole was run in parallel at a final highest concentration of 66.7 µM and 3-fold subsequent serial dilutions. The untreated negative control contained 0.14% of DMSO. Sealable test tubes filled to its maximum volume of 700 µL were used to perform this assay. The test tubes were incubated at 37 °C for 72 h. After incubation, the tubes were chilled on ice, and the reaction was transferred to 96-well plates. In the plate, 50 µL of an ATP binding luminescence marker, CellTiter-Glo (Promega, Madison, WI, USA) or 10 µL of 2.5 mg/mL d-luciferin (GoldBio, St Louis, MO, USA) was added to each well and allowed to develop at room temperature for 10 min [31,36]. The assay was read and analyzed using an EnVision Plate Reader (Perkin Elmer, Waltham, MA, USA). Response curves and EC_50_ values (the concentration at which 50% of *G. lamblia* trophozoite growth was inhibited) were calculated using GraphPad Prism (GraphPad, LaJolla, CA, USA).

**HCT-8 and HFF cytotoxicity assays:** Cytotoxicity of the compounds was determined against both confluent (the conditions of the infection assays) and growing HCT-8 and HFF host cells, as previously described [25,34]. Cells were seeded into white-sided, clear-bottom 96-well plates and grown to either 40% or 100% confluency. Compounds were added to cells at various concentrations up to 100 µM in a final DMSO concentration of 0.5%. DMSO was run in parallel as a vehicle control. After 48 h, the viability of the cells was determined by either quantification of cellular ATP (Cell-Titer Glo cell viability assay; Promega, Madison WI, USA) [34] or by MTT assay [25]. Viability was determined 24 h after compound addition to HFF cells or 48 h after compound addition to HCT-8 cells or HepG2 cells. The percent cell growth was then calculated with the following formula: % Cell Growth = [RLU_compound_/RLU_DMSO_] × 100. Samples were run in triplicate with two to three biological replicates.

**HepG2 cytotoxicity assay:** Mammalian cell cytotoxicity was assessed in HepG2 human hepatocytes using an MTS 3-(4,5-dimethylthiazol-2-yl)-5-(3-carboxymethoxyphenyl)-2-(4-sulfophenyl)-2*H*-tetrazolium)-based cytotoxicity assay. For cytotoxicity testing, ∼2250 cells were seeded into each well of a 384-well microtiter plate and incubated for 24 h. Serial dilutions of the compounds were added in triplicate starting at 25 μM. Cells were further incubated for an additional 48 h at 37 °C in an atmosphere containing 5% CO_2_. Zero-growth control wells were incubated with 5% Triton X-100 for 5 min prior to MTS addition. Following the addition of MTS to all wells, the plates were incubated for an additional 4 h under the same environmental conditions before taking absorbance measurements. Absorbance values were recorded at 490 nm using a Synergy Neo2 multimode reader (BioTek), and values were normalized based on Triton X-100-lysed and no treatment controls. Samples were run in triplicate in each assay, and the experiments were repeated three times.

***C. parvum* and *T. gondii* immunofluorescence assays (IFAs):** IFAs were conducted as previously described [37] with modifications. For *C. parvum* IFAs, HCT-8 cells were seeded on 4-well chamber cell culture glass slides (CELLTREAT Scientific Product, Pepperell, MA, USA). At confluency, the cell monolayers were infected with 5 × 10^5^ wild-type (WT) *C. parvum* oocysts prepared as previously described. Slides were spun at 1000 rpm for 3 min to allow oocysts to have full contact with the cell monolayers. At 4 hpi, 7.5 µM oidiolactone A or DMSO control, diluted in medium containing 0.6% taurocholic acid, was added to the infected cells for 20 h. At 24 hpi, the slides were fixed with 4% paraformaldehyde and permeabilized with 0.25% Triton-X. The slides were then blocked with 10% goat serum in 1x PBS. *C. parvum* intracellular stages were visualized with Sporo-glo (Waterborne inc., New Orleans, LA, USA). The slides were mounted using VECTASHIELD^®^ PLUS Antifade Mounting Medium with DAPI (Vector Laboratories, Inc., Newark CA, USA). Representative images of the samples were taken using the Nikon AX R confocal microscope and Nikon Elements acquisition software. Image processing was performed using Fiji v1.54p.

For *T. gondii* IFAs, confluent HFF cells in 4-well chamber cell culture glass slides were infected with either 5 × 10^4^ or 1 × 10^5^ parasites/mL. At 6 hpi, 7.5 µM oidiolactone A or DMSO control was diluted in culture medium and added to the slides for 24 h. The slides were then fixed with 4% paraformaldehyde and permeabilized with 0.25% Triton-X. The slides were blocked with 3% goat serum in 0.25% Triton-X. The parasites were visualized with anti-*T. gondii* SAG1 and Alexa Fluor 594 goat anti-rabbit antibody [37]. The slides were mounted, and images were acquired as described above and processed with NIS Elements GA3 (general analysis 3).

***C. parvum* excystation and invasion assays:** Oocyst excystation rates in the presence of compounds were determined as previously described [27]. The *C. parvum* invasion inhibition assays were conducted in two formats. In the first format, described previously [38], HCT-8 cells were seeded on glass slides (CELLTREAT Scientific Product, Pepperell, MA, USA) and allowed to grow to confluence. Oidiolactone A was added to the cells for one hour. TrtE and DMSO were run in parallel as positive and negative controls, respectively. The cell monolayers were then infected with 1.2 × 10^6^ wild-type *C. parvum* oocysts. After 3 h of incubation at 37 °C, the slides were washed five times with 111 mM D-galactose, fixed with 4% paraformaldehyde, and permeabilized with 0.25% Triton-X. *C. parvum* sporozoites were detected with rabbit anti-gp40 [39] and Alexa Fluor 594 goat anti-rabbit antibodies. The HCT-8 cell nuclei were stained with 0.09 mM Hoechst. Slides were coded to enable unbiased counting, and sporozoites from at least 10 randomly selected fields were counted in each sample at 100× magnification (Zeiss^®^ Axioscope 5). The percentage of invaded parasites was determined using the following equation: % Invaded parasites = [parasites_compound_/parasites_DMSO_] × 100. This experiment was run in duplicate with two biological replicates.

In the second format, HCT-8 cells were seeded onto clear-bottom, white-sided 96-well plates and grown to confluence. Compounds or DMSO were added to the cells for 1 h. The cell monolayers were then infected with 2 × 10^5^ Cp-NLuc oocysts and incubated and washed as described above. Relative luminescence was determined using the Promega Nano-Glo kit and the SpectraMax^®^ L Microplate Reader. The percentage of invaded parasites was determined using the following equation: % Invaded Parasites = [RLU_compound_/RLU_DMSO_] × 100. The experiment was run in triplicate with three biological replicates. In both experimental formats, 100 nM tartrolon E [34] was included as a positive control.

**Inhibition of *C. parvum* infection in IFN-gamma knock-out (IFNγ −/−) mice:** Twelve female C57BL/6 IFNγ −/− mice (4 weeks old) from the Jackson Laboratory were housed in a controlled environment with a 12 h light/dark cycle and access to food and water ad libitum. Cp-NLuc oocysts were isolated from infected mouse feces and purified using a sodium chloride density gradient [40]. Mice were inoculated via oral gavage with a suspension of 10^4^ oocysts in 50 µL sterile PBS. On days 5 and 6 post-infection, mice were treated with 4 mg/kg of oidiolactone A or DMSO vehicle control every 12 h for a total of four doses (6 mice per treatment group). Fecal samples were collected daily and stored at −20 °C until analysis. Feces were homogenized in a lysis buffer solution, centrifuged, and the supernatant was incubated with Nano-Glo^®^ substrate in a 96-well plate, as previously described [40]. Luminescence was measured in a microplate reader immediately after substrate addition. Oocyst shedding, measured in RLUs/gram of feces, was plotted over time, and the area under the curve was determined for each mouse. Mice were sacrificed 14 days post-infection, and serum was collected for biochemical analysis. Biochemical analyses were performed using the Beckman Coulter AU480 automated analyzer (Beckman Coulter, Brea, CA, USA) at the Clinical Pathology Laboratory, Veterinary Medical Center, University of Minnesota.

**Pharmacokinetics:**

**Plasma stability assay.** The plasma stability assay was performed in duplicate by incubating each selected compound (1 µmol/L final concentration) in normal mouse (CD-1) or human plasma (Innovative Research, Novi, MI, USA) at 37 °C. At various timepoints up to 24 h, a 40 µL aliquot of the plasma mixture was taken and quenched with 120 µL of acetonitrile containing 0.1% formic acid. The samples were then vortexed and centrifuged at 15,000 rpm (Thermo Scientific Sorvall ST 8R, Nienburg, Germany) for 5 min. The supernatants were collected and analyzed by LC-MS/MS to determine the in vitro plasma half-life (*t*_1/2_).

**Microsomal stability assay**. The in vitro microsomal stability assay was conducted in duplicate in commercially available mouse or human liver microsomes (Sekisui XenoTech, Kansas City, KS, USA), which were supplemented with nicotinamide adenine dinucleotide phosphate (NADPH) as a cofactor. Briefly, a compound (1 µmol/L final concentration) was spiked into the reaction mixture containing liver microsomal protein (0.5 mg/mL final concentration) and MgCl_2_ (1 mmol/L final concentration) in 0.1 mol/L potassium phosphate buffer (pH 7.4). The reaction was initiated by the addition of 1 mmol/L NADPH, followed by incubation at 37 °C. A negative control was performed in parallel without NADPH to reveal any chemical instability or non-NADPH dependent enzymatic degradation for each compound. A reaction with positive control verapamil was also performed as in-house quality control to confirm the proper functionality of the incubation systems. At various time points (0, 5, 15, 30, and 60 min), a 40 µL of reaction aliquot was taken and quenched with 120 µL of acetonitrile containing 0.1% formic acid. The samples were then vortexed and centrifuged at 15,000 rpm for 5 min at 4 °C. The supernatants were collected and analyzed by LC-MS/MS to determine the in vitro metabolic half-life (*t*_1/2_).

**PAMPA membrane permeability assay.** The membrane permeability of selected compounds was evaluated using the Corning^®^ BioCoat^TM^ Pre-coated PAMPA Plate System (catalog. No. 353015, Corning, Glendale, AZ, USA). The pre-coated plate assembly, which was stored at −20 °C, was thawed for 30 min at room temperature. The permeability assay was carried out in accordance with the manufacturer’s protocol. Briefly, the 96-well filter plate, pre-coated with lipids, was used as the permeation acceptor and a matching 96-well receiver plate was used as the permeation donor. Compound solutions were prepared by diluting the 10 mmol/L DMSO stock solutions with DPBS to a final concentration of 100 μmol/L. The compound solutions were added to the wells (300 μL/well) of the receiver plate, and DPBS was added to the wells (200 μL/well) of the pre-coated filter plate. The filter plate was then coupled with the receiver plate, and the plate assembly was incubated at 25 °C without agitation for 5 h. At the end of the incubation, the plate was separated, and the final concentrations of compounds in both donor wells and acceptor wells were analyzed using LC-MS/MS. Permeability of the compounds was calculated using Equation (1):

*Pe =* {−*ln* [*1* − *C_A_*(*t*)/*C_eq_*]}/[*A* × (*1*/*V_D_* + *1*/*V_A_*) × *t*] (1)

where *A* = filter area (0.3 cm^2^), V_D_ = donor well volume (0.3 mL), V_A_ = acceptor well volume (0.2 mL), *t* = incubation time (seconds), C_A_(*t*) = compound concentration in acceptor well at time *t*, and C_D_(*t*) = compound concentration in donor well at time *t*. C_eq_ was calculated using Equation (2):

*C_eq_* = [*C_D_*(*t*) × *V_D_* + *C_A_*(*t*) × *V_A_*]/(*V_D_* + *V_A_*) (2)

A cutoff criteria of *Pe* value at 1.5 × 10^−6^ cm/s was used to classify the compounds into high and low permeability according to the literature report of this PAMPA plate system [41].

The logP and logS values were calculated in silico using the SwissADME server [42], and analysis of P-gp substrate/inhibitor likelihood was determined using the PgpRules prediction server [43].

**Statistics:** The inhibition curves and EC_50_s were determined using the log [inhibitor] vs. response-variable slope (four-parameter) regression equation of GraphPad Prism v10.0.1. Differences between groups were determined by parametric or non-parametric *t*-tests, as indicated in figure legends. Results were considered statistically significant at *p* < 0.05.

## 3. Results

### 3.1. Two Norditerpene Lactones Exhibit Potent Activity Against C. parvum and T. gondii

**Figure 1:**
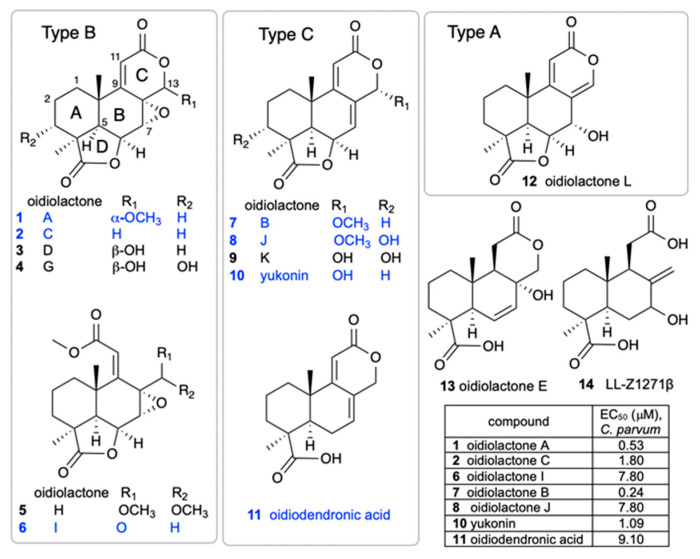
Structures of oidiolactone norditerpenes (**1**–**14**) tested against intracellular, nanoluciferase-expressing *C. parvum* in vitro. Active compounds are indicated in blue, and EC_50_ (µM) values are shown in the table. The EC_50_s for inactive compounds (black) were not determined.

A small library of 192 extracts, enriched fractions, and pure compounds from 23 fungi and 1 bacterial species isolated from the Soudan Iron Mine, in Tower, Minnesota, was screened in an initial pilot assay [33] against CpNLuc-infected HCT-8 cells at an initial dose of 5 µg/mL. A series of seven structurally related norditerpenes isolated from the fungus *O. truncatum* showed 80–96% parasite inhibition, while seven additional congeners were inactive (Figure 1). The active compounds were retested at several concentrations to compare potencies (Figure 1, table). The two most active compounds were oidiolactones A (**1**) and B (**7**).

### 3.2. Oidiolactones A and B Exhibit Anti-Apicomplexan Activity Without Toxicity to Host Cells

**Figure 2:**
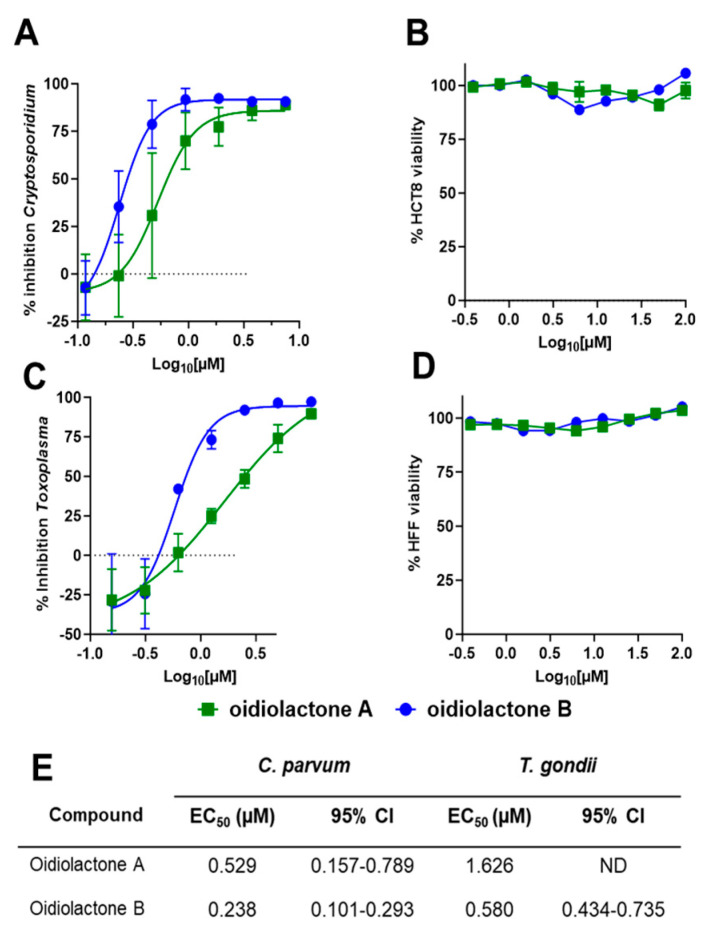
The oidiolactones exhibit specific nM-level activity against *C. parvum* and *T. gondii*. Compounds were tested at concentrations ranging from 7.5 µM to 117 nM against Cp-NLuc-infected HCT-8 cells (**A**) or 10 µM to 156 nM against TgRH-Luc:GFP-infected HFF cells (**C**). Results are from 3 biological replicates. Confluent HCT-8 (**B**) and HFF (**D**) cells were incubated with compounds (100 µM to 390 nM) for 48 h or 24 h, respectively. Cell viability was determined by quantification of cellular ATP levels. Results are from 2 biological replicates. Error bars show the standard deviation around each point in (**A**–**D**). (**E**) Summary of EC_50_s and confidence intervals. EC_50_s were determined in Graphpad Prism using the log (inhibitor) vs. response–variable slope (four parameters).

Oidiolactones A and B were tested against HCT-8 infected Cp-NLuc using an expanded range of concentrations to calculate an accurate EC_50_ (Figure 2A). These select compounds were also tested against fibroblasts infected with the related apicomplexan parasite *Toxoplasma gondii* (TgRH-Luc:GFP; Figure 2C). The EC_50_s of oidiolactones A and B against intracellular Cp-NLuc and TgRH-Luc:GFP were in the submicromolar range, with oidiolactone B twice as potent as oidiolactone A against both parasites (Figure 2E). Neither compound showed any toxicity to host cells up to 100 µM when tested under the conditions of the infection assay (Figure 2B,D).

Since the compounds exhibited activity against two divergent apicomplexan parasites, the seven bioactive oidiolactones (Figure 1) were also tested for activity against *P. falciparum,* the causative agent of malaria. Only oidiolactones A and B had anti-*P. falciparum* activity but at low micromolar concentrations (Supplementary Table S1). We further tested oidiolactones A and B against *G. lamblia,* an infectious protozoan that is phylogenetically distant from the apicomplexan parasites, to investigate potential broader spectrum antiparasitic activities of these inhibitors. We found that oidiolactones A and B had no activity (up to 29 µM) against this protozoan parasite (Supplementary Figure S1).

### 3.3. Oidiolactone B Exhibits Some Cytotoxicity Towards Sub-Confluent HCT-8 Cells

To see if the compounds had any effects on actively growing cells, we tested oidiolactones A and B against sub-confluent host cells (~40% confluency). Both oidiolactones A and B exhibited some cytotoxicity against sub-confluent HCT-8 cells (with EC_50_s of 29.7 µM and 2.1 µM, respectively; Supplementary Table S2). There was no cytotoxicity observed against sub-confluent HFF (Supplementary Table S2), human fibroblast [25], or HepG2 cells (Supplementary Table S1).

### 3.4. Structure–Activity Relationship of C. parvum Inhibition and HCT-8 Cytotoxicity

The isolation of a suite of related norditerpenes with a wide range of anti-parasitic and cytotoxic activities allowed us to identify some structural features correlated with biological activity. The tetracyclic norditerpene dilactones produced by fungi and plants are generally classified into three structural groups (Figure 1). Type A norditerpenes contain an ɑ-pyrone [8(14), 9(11)-dieneone], type B compounds include a 7ɑ, 8ɑ-epoxy-9(11)-dieneone, and type C compounds have a 7(8), 9(11)-dieneone structure. Among the group of 14 *O. truncatum* compounds that we tested, there were examples in all three structural classes (albeit only a single “type A” example). We also want to emphasize the confusing nature of the trivial names of these compounds compared with their structure class (e.g. oidiolactones A and C are “type B” norditerpenes, while oidiolactone B is a “type A” norditerpene), along with references to rings A through D.

Comparison of the two most active compounds oidiolactones A (**1**) and B (**7**), shows that the presence of a 7,8 double bond vs. a 7ɑ, 8ɑ-epoxy group leads to a 2-fold increase in inhibitory activity against *C. parvum* and 2.6-fold increase against *T. gondii* (Figure 2E). However, this structural difference also causes oidolactone B to be ~15x more cytotoxic towards the host HCT-8 cells under non-confluent conditions (Supplementary Table S2). An ɑ-OMe group at C-13 vs. a hydroxy moiety also leads to higher levels of anti-parasitic activity (compare **1** to **3** and **7** to **10**). The presence of a hydroxy group on C-3 of the A ring eliminates activity for the epoxide derivatives (compare **1** to **4**) and significantly decreases parasite growth inhibition for the type C dieneones (compare **7** to **8**, and **10** to **9**). Opening of the δ-lactone ring (ring C) abolishes inhibitory activity for compound **5** with geminal O-methyl groups and reduces activity for the only aldehyde derivative in the series, **6**. Opening of the *γ*-lactone (ring D, compound **11**) strongly reduces activity. The only example of an ɑ-pyrone norditerpene in this series, compound **12**, was inactive against the parasites (Figure 1) and lacked cytotoxicity (Supplementary Table S2 and [25]).

Although oidiolactone B (**7**) was the most potent inhibitor of *C. parvum* in this series, oidiolactone A (**1**) had a higher therapeutic index when comparing the sub-confluent HCT-8 cytotoxicities (TI: EC_50_ *C. parvum*/EC_50_ HCT-8 = 56). In further characterizing anti-parasitic activity in vitro and in vivo, we therefore focused on oidiolactone A as the most potent and potentially least cytotoxic compound.

### 3.5. In Vitro Pharmacokinetics of Oidiolactone A

There is no consensus about whether oral *Cryptosporidium* drugs should target intracellular (but extra-cytoplasmic) parasites in the gastrointestinal lumen directly or be absorbed systemically (i.e., have high oral bioavailability). We assessed the in vitro stability of oidiolactone A with both human and mouse blood plasma and liver microsomes to identify potential liabilities and help predict its in vivo pharmacokinetics and develop a preliminary dosing regimen for in vivo studies. The compound was less stable in human vs. mouse plasma, in contrast to most reports of small-molecule in vitro stability comparisons across species [44], but more stable in human vs. mouse liver microsomes (Table 1). We also determined that oidiolactone A exhibits high passive permeability using PAMPA (Pe 11.0 ± 1.3 × 10^−6^ cm/s), which is consistent with the calculated lipophilicity and solubility values (Log *p* = 1.44 and Log S = 2.46). Oidiolactone A is not predicted to be a substrate or inhibitor of p-glycoprotein receptors [43].

### 3.6. Oidiolactone A Inhibits Intracellular C. parvum and T. gondii Replication

**Figure 3:**
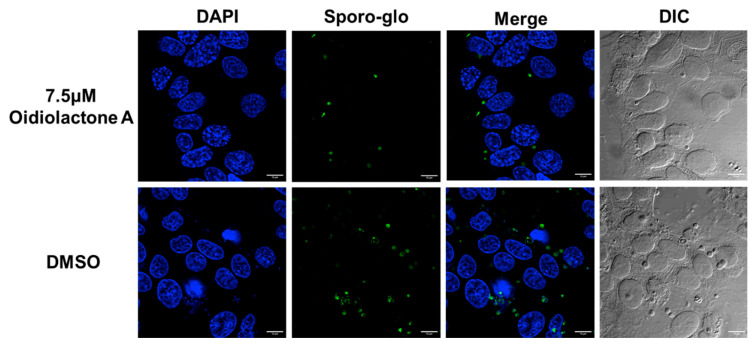
Oidiolactone A inhibits growth of *Cryptosporidium parvum*. HCT-8 cells infected with wild-type *C. parvum* for 4 h were treated with oidiolactone A for 20 h. Cells were then fixed and labeled with Sporoglo (green) to visualize the intracellular meronts and extracellular zoites. Host cell nuclei were labeled with Hoescht (blue). Scale bar = 10 µm.

**Figure 4:**
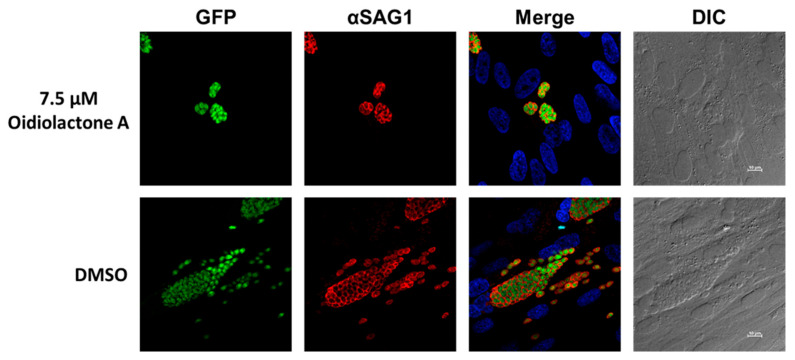
Oidiolactone A inhibits growth of *Toxoplasma gondii*. TgRH-Luc:GFP infected HFFs were treated with oidiolactone A for 24 h. Cells were then fixed and labeled with anti-SAG1 antibody (red) to visualize the tachyzoite membrane and Hoescht (blue) to label host cell nuclei. Scale bar = 10 µm.

To determine if oidiolactone A reduced the number of replicating intracellular parasites, treated parasites were evaluated by IFA. Intracellular parasites were treated for 20–24 h with oidiolactone A or DMSO control, fixed, and then visualized with either Sporoglo for *Cryptosporidium* (Figure 3; Supplementary Figure S2) or anti- SAG1 antibody for *Toxoplasma* (Figure 4; Supplementary Figure S3). Treated parasites were present at much lower abundances (Figure 3 and Figure 4; Supplementary Figures S2 and S3), consistent with the results from infection inhibition assays (Figure 2A,C).

### 3.7. Oidiolactone A Has -Cidal Activity Against Both C. parvum and T. gondii

**Figure 5:**
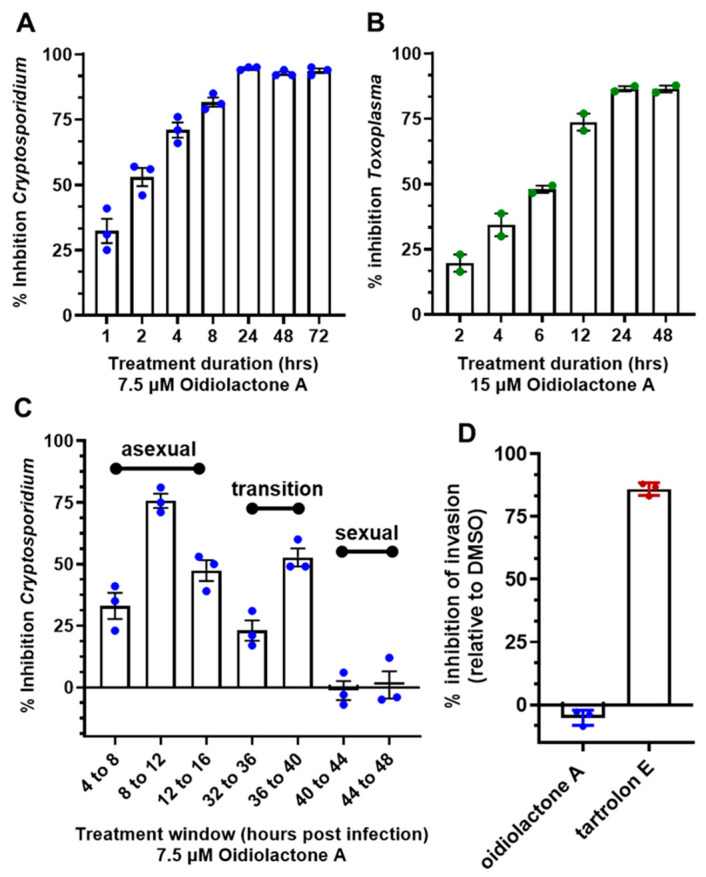
Oidiolactone A rapidly kills intracellular, asexual parasite stages, but does not inhibit sporozoite invasion into host cells. (**A**) Cp-NLuc was allowed to infect HCT-8 cells for 24 h, at which point compound or DMSO vehicle control was added for the lengths of time indicated. At the end of each time point, the compound was washed out and infection was allowed to proceed until the plate was read at 72 h post-infection. Results are the percent of growth inhibition relative to the DMSO control. (**B**) HFF cells infected with TgRH-Luc:GFP parasites were treated as described in (**A**), but the plate was read 48 h post-infection. Results calculated as in (**A**). (**C**) HCT-8 cells were infected with Cp-NLuc, and the compound or DMSO added for 4 h intervals, as indicated. The plate was read at 72 h post-infection, and percent inhibition of parasite growth was calculated based on DMSO control for that time interval. (**D**) Cp-NLuc oocysts were added to HCT-8 cells incubated in 7.5 µM oidiolactone A, 100 nM tartrolon E, or DMSO and allowed to infect for 3 h, at which point plate was read for luciferase expression. Percent inhibition was calculated by comparison to DMSO control. All data were compiled from 3 biological replicates. Error bars: +/−SD.

Since oidiolactone A inhibited parasite growth but did not appear to overtly damage parasites (Figure 3 and Figure 4; Supplementary Figures S2 and S3), intracellular Cp-NLuc and TgRH-Luc:GFP parasites were treated for varying lengths of time, from 4 to 24 h, after which the compound was removed and the parasites were allowed to recover for a further 48 h for Cp-NLuc and 24 h for TgRH-Luc:GFP. After 8–12 h of treatment with oidiolactone A at the EC_90_ (7.5 µM for Cp-NLuc and 15 µM for TgRH-Luc:GFP), neither Cp-NLuc or TgRH-Luc:GFP recovered, suggesting a -cidal versus -static effect (Figure 5A and Figure 5B, respectively).

### 3.8. Oidiolactone A Inhibits C. parvum Parasites During Asexual Replication and the Transition to Sexual Stages

While *T. gondii* tachyzoite cultures, used here to determine anti-*Toxoplasma* activity, are representative of just the one life cycle stage, *C. parvum* in vitro cultures develop through three rounds of asexual replication during the first ~32 h, at which time they begin to transition to formation of the male and female gametes [4]. By 44 h post-infection, the culture consists of primarily gametes. In standard 2D cultures, the life cycle halts at fertilization and no oocysts are produced. However, the accurate timing of asexual and sexual development in HCT-8 cells has been reported [4], and this timeline can be used to evaluate the susceptibility of different parasite stages to anti-parasitic compounds [4,45]. Oidiolactone A (7.5 μM) was added to HCT-8 cells for 4 h intervals post-infection with NLuc-*C. parvum* oocysts. Inhibition (20–75%) was observed only for parasites in the asexual stage of proliferation, and during transition to sexual stages (Figure 5C). Oidiolactone A exhibited no activity against gamete stages.

### 3.9. Oidiolactone A Does Not Inhibit Excystation or Prevent Invasion of C. parvum Sporozoites into Host Cells

Compounds that can prevent excystation or sporozoite invasion into host cells could potentially be used as prophylactic agents, an application critical to prevent calf cryptosporidiosis and subsequent environmental contamination. Oidiolactone A had no effect on oocyst excystation (Supplementary Figure S4A). The ability of oidiolactone A to inhibit invasion was evaluated by luciferase expression (Figure 5D) and by immunofluorescence assay with enumeration of trophozoite stages (Supplementary Figure S4B). Oidiolactone A did not exhibit any effect on parasite invasion, in contrast to the control compound tartrolon E [34], which completely inhibited parasite invasion (Figure 5D).

### 3.10. Oidiolactone A Reduces Infection in IFNγ−/− Mice Infected with Cp-NLuc

**Figure 6:**
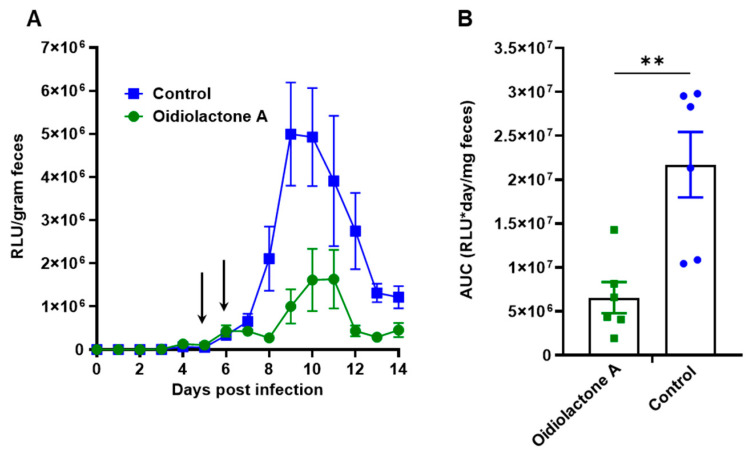
Oidiolactone A significantly reduces oocyst shedding in *C. parvum*-infected IFNγ-knockout mice. (**A**) Mice were infected with Cp-NLuc, and oocyst shedding was monitored daily by luciferase expression. On days 5 and 6 post-infection, 4 mg/kg oidiolactone A was administered every 12 h by oral gavage (arrows). Controls received DMSO in parallel. Error bars represent standard error of the mean. (**B**) The area under the curve (AUC) of the oocyst shedding for each mouse was calculated. Each point is data from one mouse. A Mann–Whitney *t*-test was used to compare the groups. ** *p* = 0.0087.

We evaluated the in vivo efficacy of oidiolactone A in a model of acute *C. parvum* infection in mice that lack IFNγ. Five-week-old IFNγ−/− mice were infected with mouse-adapted Cp-NLuc oocysts. On days 5 and 6 post-infection, mice were treated with four doses of 4 mg/kg of oidiolactone A by oral gavage, every 12 h. Control mice were treated in parallel with the vehicle control (DMSO). Fecal samples were collected daily, until day 14 post-infection, when the mice were euthanized, and serum was harvested for biochemical profiling. Over the course of infection, oidiolactone A reduced shedding of oocysts, as determined by RLUs per gram of feces, (Figure 6A) indicating that the treatment with oidiolactone A was effective in reducing the parasite load. The treatment resulted in an overall 70% reduction in *C. parvum* oocyst shedding (Figure 6B), suggesting that this compound has therapeutic potential for treating cryptosporidiosis. Additionally, assessments of liver, renal, metabolic, and cardiac functions, as well as protein status and electrolyte levels, showed no statistically significant differences between the control and treated groups, indicating the absence of any signs of acute toxicity (Supplementary Table S3).

## 4. Discussion

*Cryptosporidium* remains one of the few, if not the only, globally distributed pathogens with no effective vaccines or therapeutics for prevention and treatment [46]. To date, the number of compounds in the therapeutic pipeline for cryptosporidiosis is limited, and the most promising target is a single enzyme, raising the possibility that resistance could arise once these compounds are used clinically [14]. Several compounds are under advanced preclinical or clinical investigation [34,47,48,49,50,51,52,53,54]. A repurposed leprosy drug, clofazimine, with anti-Cryptosporidium activity, failed in clinical trials due to solubility and bioavailability issues [55], highlighting the need for early pharmacokinetic analysis and multiple lead compounds. Very little investigation into natural products as a source of anti-*Cryptosporidium* compounds has been conducted, despite the fact that parasitic gregarines, close relatives of *Cryptosporidium*, are widely found in all environments [56] and may provide an ecological basis for the evolution of natural anti-parasitics [34]. Additionally, natural products have historically provided us with our most effective and robust anti-parasitic drugs [23,24]. Thus far, two purified natural compounds have been reported to have anti-*Cryptosporidium* activity. One of these, tartrolon E, isolated from a shipworm bacterial symbiont, has rapid killing activity against the parasite, is highly effective in vivo, and is also broadly effective against several apicomplexan parasites [34]. The marine sponge metabolite, leiodolide A, has potent submicromolar in vitro activity against *Cryptosporidium*, underscoring the potential of natural products as a source of therapeutics for cryptosporidiosis [33]. This report of norditerpene oidiolactones with anti-parasitic activity, identified in a very small phenotypic screen, further supports this approach.

Oidiolactone norditerpenes are members of a large, diverse group of tetracyclic dilactone terpenoid metabolites isolated from several fungal species and more than a dozen plant species in the coniferous Podocarpaceae family [57,58,59]. Some of the plant-derived compounds have been studied for their potential ecological roles as insecticides, anti-feedants, herbicides, and plant growth regulators [60,61,62]. Many of the fungal and plant norditerpenes have also been tested for potential medicinal applications and have been reported to have broad anti-microbial activities (bacteria, fungal, and parasitic), as well as anti-oncogenic and anti-inflammatory activity [59]. This study is the first report of members of this structural class having potent activity against the apicomplexan parasites *C. parvum* and *T. gondii.* Seven structurally related norditerpene lactones (=oidiolactones) with antiparasitic activity were identified from an initial in vitro growth inhibition screen. Both oidiolactones A and B had submicromolar EC_50_s against intracellular *C. parvum* and *T. gondii*, with little cytotoxicity for mammalian cells. Since oidiolactone A exhibited less toxicity towards growing HCT-8 cells, further investigation of the anti-parasitic activity of the fungal norditerpenes was conducted with oidiolactone A.

Oidiolactone A killed replicating *T. gondii* and *C. parvum* within 24 h and was primarily effective against asexual stages of *C. parvum*. The compound did not inhibit sporozoite infection of host cells. Most critically, it was highly effective against the parasite in vivo in an immunocompromised mouse model of acute infection, even at a low dose (4 mg/kg). To evaluate the range of activity against other protozoan parasites, we also tested the compounds against the causative agent of malaria, *P. falciparum,* and the intestinal parasite *G. lamblia*. Oidiolactone A exhibited low micromolar activity against *P. falciparum* but did not inhibit *G. lamblia* (EC_50_ > 30 μM).

There is no clear agreement on the ideal pharmacokinetic parameters for effective anti-cryptosporidial drugs. There is some data suggesting that optimal anti-cryptosporidial compounds would be retained in the gut with slow systemic uptake [63,64,65]. For example, the in vivo efficacy of a series of related bumped kinase inhibitor (BKI) pre-clinical lead compounds, ATP-competitive inhibitors of parasite calcium-dependent protein kinases, was found to depend solely on their concentration in the large intestine [65]. It is unclear if this would be true for all classes of anti-*Cryptosporidium* compounds. Retention in the intestine minimizes the need for plasma and microsomal stability and high bioavailability, and potentially reduces off-target cytotoxicity effects [63]. The relatively short half-life of oidiolactone A in mouse blood plasma (<2 h), high membrane permeability, and in vivo efficacy suggests that the compound may act through direct exposure in the intestine. An additional consideration for intestinal absorption of drugs is the involvement of P-glycoprotein (P-gp) located on the apical membrane of enterocytes in the intestinal epithelium [66]. These receptors function to pump substrates back into the intestinal lumen, effectively reducing their intracellular concentration and systemic absorption. Recent research with the BKIs found that derivatives that were not recognized by P-gp were more effective in vivo [67]. Oidiolactone A is not predicted to be a P-gp substrate or inhibitor [43,68], but additional testing using in vitro bidirectional permeability assays and co-administration studies with P-gp inhibitors will be needed for confirmation [67,69].

**Figure 7:**
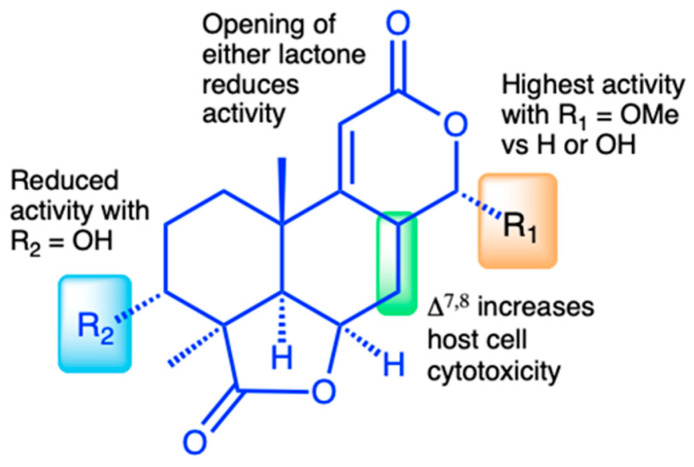
Structure–activity relationship (SAR) summary for oidiolactones from *O. truncatum* tested against NLuc *C. parvum* in HCT-8 cells. “Activity” refers to inhibition of parasite growth, and “cytotoxicity” refers to inhibition of host cell growth under sub-confluent conditions.

The discovery of a suite of 14 structurally related norditerpene lactone derivatives with widely different anti-parasitic activities provided an opportunity to compare the structure–activity relationships (SARs) related to both anti-parasitic activity and host cell cytotoxicity (Figure 7). For this series, the primary features of the most active compounds include a C-13 O-methyl ether, lack of a hydroxy group at C-3, and intact lactone rings. Although the type C diene congener, oidiolactone B, was the most potent inhibitor of *C. parvum* and *T. gondii*, it was also more toxic towards actively dividing host cells, while the type B epoxide derivative, oidiolactone A, had a higher therapeutic index. This initial set of SAR data provides a useful set of guidelines for further testing and discovery of additional related natural norditerpene dilactones, as well as synthetic derivatives.

Oidiolactone A is also an attractive compound for optimization, as it is produced in high concentrations by the fungus (~60 mg/L) under unoptimized conditions and has several functional group “handles” for semi-synthetic transformations. Because there has been significant interest in the potential anti-cancer activity of many norditerpene dilactones, such as the nagilactones and podolactones, there is also a rich literature precedent for both semi-synthesis and de novo synthesis for several members of this class [70,71,72], providing established methods for production or modification. Additionally, several of the anti-cancer studies with related norditerpene dilactones included both oral and intraperitoneal administration of candidate compounds using mouse models, which provides some additional data about in vivo dosing, oral bioavailability, and toxicity [73,74,75].

Although the molecular target is not yet known, it is likely that the mechanism of action of the norditerpene dilactone metabolites is different than for other known cryptosporidial inhibitors because the structure is so distinct from reported anti-*Cryptosporidium* compounds [34,47,48,49,50,51,52,53,54,55,56,57]. Furthermore, the lack of phenotypic suppression of *G*. *lamblia* growth but potent inhibition of *C. parvum*, *T. gondii*, and *P. falciparum* proliferation suggests that the primary mechanism of action may be unique to apicomplexan protozoan parasites biology. Previously identified targets of oidiolactones and closely related analogs associated with their anti-cancer activities include IL-1β and TNF-ɑ [76], while the nagilactones and podolactones were found to induce apoptosis [74], inhibit protein synthesis [77], and inhibit transcription factor AP-1, activator protein 1 [78,79]. Oidiolactone A inhibits intracellular replication of the parasites. It is unclear if this activity is due to the inhibition of a parasite-specific target or modulation of a host cell factor or process that the parasite requires for intracellular growth.

Oidiolactone A demonstrates strong anti-cryptosporidial activity both in vitro and in vivo and is readily accessible as a natural product and via well-established synthetic methods. The results from these preliminary assessments suggest that the norditerpene dilactone pharmacophophore is a promising new lead for antiparasitic drug development and provides a clear rationale for the testing of additional natural and semi-synthetic derivatives to identify more potent and effective compounds. These initial experiments indicate that oidiolactone A is effective as a short-dose oral treatment in an acute mouse model of cryptosporidiosis, but it is not yet known if it would reduce parasites in an extended chronic model of infection, which will be important for future studies. Collectively, these data provide compelling evidence of the potential of this class of norditerpenes as therapeutics for untreatable cryptosporidiosis.

## Figures and Tables

**Table 1:** In vitro metabolic stabilities of oidolactone A.

	Mouse Plasma Stability t½ (min)	Human Plasma Stability t½ (min)	Mouse Microsomal Stability ^a^ t½ (min)	Human Microsomal Stability t½ (min)
Oidiolactone A (1)	105.8 ± 1.7	17.5 ± 0.1	110.4 ± 4.3 ^b^	317.7 ± 16.7
Verapamil			2.8 ± 0.0	11.2 ± 0.2

Data are presented as mean ± SD. ^a^ CYP enzyme cofactor: NADPH. ^b^ Control in the absence of NADPH showed remaining percentage less than 80% at the end of incubation of 60 min (susceptible to non-NADPH-dependent degradation).

## Data Availability

The raw data supporting the conclusions of this article will be made available by the authors on request.

## Supplementary Materials

The following supporting information can be downloaded at https://www.mdpi.com/article/10.3390/microorganisms13112527/s1: Table S1: Activity of oidiolactones against *Plasmodium falciparum* isolates and HepG2 cells; Table S2: Cytotoxicity of oidiolactones against confluent and sub-confluent host cells; Table S3: Biochemical analysis of serum from infected and treated IFNγ −/− mice; Figure S1: Dose–response curves of oidiolactones A and B against *G. lamblia*; Figure S2: *C. parvum* intracellular stages treated with oidiolactone A; Figure S3: *T. gondii* intracellular stages treated with oidiolactone A.; Figure S4: Oidiolactone A has no effect on oocyst excystation or sporozoites invasion into host cells.

## References

[1] Ma J.-Y., Li M.-Y., Qi Z.-Z., Fu M., Sun T.-F., Elsheikha H.M., Cong W. (2022). Waterborne Protozoan Outbreaks: An Update on the Global, Regional, and National Prevalence from 2017 to 2020 and Sources of Contamination. Sci. Total Environ..

[2] Ali M., Ji Y., Xu C., Hina Q., Javed U., Li K. (2024). Food and Waterborne Cryptosporidiosis from a One Health Perspective: A Comprehensive Review. Animals.

[3] Tzipori S., Ward H. (2002). Cryptosporidiosis: Biology, Pathogenesis and Disease. Microbes Infect..

[4] English E.D., Guérin A., Tandel J., Striepen B. (2022). Live Imaging of the *Cryptosporidium parvum* Life Cycle Reveals Direct Development of Male and Female Gametes from Type I Meronts. PLoS Biol..

[5] Checkley W., White A.C., Jaganath D., Arrowood M.J., Chalmers R.M., Chen X.-M., Fayer R., Griffiths J.K., Guerrant R.L., Hedstrom L. (2015). A Review of the Global Burden, Novel Diagnostics, Therapeutics, and Vaccine Targets for *Cryptosporidium*. Lancet Infect. Dis..

[6] Mmbaga B.T., Houpt E.R. (2017). *Cryptosporidium* and *Giardia* Infections in Children: A Review. Pediatr. Clin. N. Am..

[7] Mac Kenzie W.R., Hoxie N.J., Proctor M.E., Gradus M.S., Blair K.A., Peterson D.E., Kazmierczak J.J., Addiss D.G., Fox K.R., Rose J.B. (1994). A massive outbreak in Milwaukee of cryptosporidium infection transmitted through the public water supply. N. Engl. J. Med..

[8] Bourli P., Eslahi A.V., Tzoraki O., Karanis P. (2023). Waterborne Transmission of Protozoan Parasites: A Review of Worldwide Outbreaks—An Update 2017–2022. J. Water Health.

[9] Wang R.-J., Li J.-Q., Chen Y.-C., Zhang L.-X., Xiao L.-H. (2018). Widespread Occurrence of *Cryptosporidium* Infections in Patients with HIV/AIDS: Epidemiology, Clinical Feature, Diagnosis, and Therapy. Acta Trop..

[10] Kotloff K.L., Nataro J.P., Blackwelder W.C., Nasrin D., Farag T.H., Panchalingam S., Wu Y., Sow S.O., Sur D., Breiman R.F. (2013). Burden and Aetiology of Diarrhoeal Disease in Infants and Young Children in Developing Countries (the Global Enteric Multicenter Study, GEMS): A Prospective, Case-Control Study. Lancet.

[11] Abubakar I.I., Aliyu S.H., Arumugam C., Hunter P.R., Usman N. (2007). Prevention and Treatment of Cryptosporidiosis in Immunocompromised Patients. Cochrane Libr..

[12] Tam P., Arnold S.L.M., Barrett L.K., Chen C.R., Conrad T.M., Douglas E., Gordon M.A., Hebert D., Henrion M., Hermann D. (2021). Clofazimine for Treatment of Cryptosporidiosis in Human Immunodeficiency Virus Infected Adults: An Experimental Medicine, Randomized, Double-Blind, Placebo-Controlled Phase 2a Trial. Clin. Infect. Dis..

[13] Khan S.M., Witola W.H. (2023). Past, Current, and Potential Treatments for Cryptosporidiosis in Humans and Farm Animals: A Comprehensive Review. Front. Cell. Infect. Microbiol..

[14] Love M.S., Choy R.K.M. (2021). Emerging Treatment Options for Cryptosporidiosis. Curr. Opin. Infect. Dis..

[15] Shaw H.J., Innes E.A., Morrison L.J., Katzer F., Wells B. (2020). Long-Term Production Effects of Clinical Cryptosporidiosis in Neonatal Calves. Int. J. Parasitol..

[16] Santín M. (2013). Clinical and Subclinical Infections with *Cryptosporidium* in Animals. N. Z. Vet. J..

[17] Thomson S., Hamilton C.A., Hope J.C., Katzer F., Mabbott N.A., Morrison L.J., Innes E.A. (2017). Bovine Cryptosporidiosis: Impact, Host-Parasite Interaction and Control Strategies. Vet. Res..

[18] Porras G., Chassagne F., Lyles J.T., Marquez L., Dettweiler M., Salam A.M., Samarakoon T., Shabih S., Farrokhi D.R., Quave C.L. (2021). Ethnobotany and the Role of Plant Natural Products in Antibiotic Drug Discovery. Chem. Rev..

[19] Christensen S.B. (2021). Natural Products That Changed Society. Biomedicines.

[20] Tu Y. (2011). The Discovery of Artemisinin (Qinghaosu) and Gifts from Chinese Medicine. Nat. Med..

[21] Burg R.W., Miller B.M., Baker E.E., Birnbaum J., Currie S.A., Hartman R., Kong Y.L., Monaghan R.L., Olson G., Putter I. (1979). Avermectins, New Family of Potent Anthelmintic Agents: Producing Organism and Fermentation. Antimicrob. Agents Chemother..

[22] Chabala J.C., Mrozik H., Tolman R.L., Eskola P., Lusi A., Peterson L.H., Woods M.F., Fisher M.H., Campbell W.C., Egerton J.R. (1980). Ivermectin, a New Broad-Spectrum Antiparasitic Agent. J. Med. Chem..

[23] Hertweck C. (2015). Natural Products as Source of Therapeutics against Parasitic Diseases. Angew. Chem. Int. Ed. Engl..

[24] Ndjonka D., Rapado L.N., Silber A.M., Liebau E., Wrenger C. (2013). Natural Products as a Source for Treating Neglected Parasitic Diseases. Int. J. Mol. Sci..

[25] Rusman Y., Wilson M.B., Williams J.M., Held B.W., Blanchette R.A., Anderson B.N., Lupfer C.R., Salomon C.E. (2020). Antifungal Norditerpene Oidiolactones from the Fungus *Oidiodendron truncatum*, a Potential Biocontrol Agent for White-Nose Syndrome in Bats. J. Nat. Prod..

[26] Pettit G.R., Tan R., Herald D.L., Hamblin J., Pettit R.K. (2003). Antineoplastic Agents. 488. Isolation and Structure of Yukonin from a Yukon Territory Fungus. J. Nat. Prod..

[27] Cotto-Rosario A., Miller E.Y.D., Fumuso F.G., Clement J.A., Todd M.J., O’Connor R.M. (2022). The Marine Compound Tartrolon E Targets the Asexual and Early Sexual Stages of *Cryptosporidium parvum*. Microorganisms.

[28] Roos D.S., Donald R.G., Morrissette N.S., Moulton A.L. (1994). Molecular Tools for Genetic Dissection of the Protozoan Parasite *Toxoplasma gondii*. Methods Cell Biol..

[29] Trager W., Jensen J.B. (1976). Human Malaria Parasites in Continuous Culture. Science.

[30] Nardella F., Jiang T., Wang L., Bohmer M.J., Chakraborty S., Okombo J., Calla J., Silva T.M., Pazicky S., Che J. (2025). *Plasmodium falciparum* Protein Kinase 6 and Hemozoin Formation Are Inhibited by a Type II Human Kinase Inhibitor Exhibiting Antimalarial Activity. Cell Chem. Biol..

[31] Michaels S.A., Shih H.-W., Zhang B., Navaluna E.D., Zhang Z., Ranade R.M., Gillespie J.R., Merritt E.A., Fan E., Buckner F.S. (2020). Methionyl-TRNA Synthetase Inhibitor Has Potent in Vivo Activity in a Novel *Giardia lamblia* Luciferase Murine Infection Model. J. Antimicrob. Chemother..

[32] Hulverson M.A., Michaels S.A., Lee J.W., Wendt K.L., Tran L.T., Choi R., Van Voorhis W.C., Cichewicz R.H., Ojo K.K. (2023). Identification of Fungus-Derived Natural Products as New Antigiardial Scaffolds. Microbiol. Spectr..

[33] Bone Relat R.M., Winder P.L., Bowden G.D., Guzmán E.A., Peterson T.A., Pomponi S.A., Roberts J.C., Wright A.E., O’Connor R.M. (2022). High-Throughput Screening of a Marine Compound Library Identifies Anti-*Cryptosporidium* Activity of Leiodolide A. Mar. Drugs.

[34] O’Connor R.M., Nepveux V F.J., Abenoja J., Bowden G., Reis P., Beaushaw J., Bone Relat R.M., Driskell I., Gimenez F., Riggs M.W. (2020). A Symbiotic Bacterium of Shipworms Produces a Compound with Broad Spectrum Anti-Apicomplexan Activity. PLoS Pathog..

[35] Smilkstein M., Sriwilaijaroen N., Kelly J.X., Wilairat P., Riscoe M. (2004). Simple and Inexpensive Fluorescence-Based Technique for High-Throughput Antimalarial Drug Screening. Antimicrob. Agents Chemother..

[36] Michaels S.A., Hulverson M.A., Whitman G.R., Tran L.T., Choi R., Fan E., McNamara C.W., Love M.S., Ojo K.K. (2022). Repurposing the Kinase Inhibitor Mavelertinib for Giardiasis Therapy. Antimicrob. Agents Chemother..

[37] O’Connor R.M., Kim K., Khan F., Ward H.D. (2003). Expression of Cpgp40/15 in *Toxoplasma gondii*: A Surrogate System for the Study of *Cryptosporidium* Glycoprotein Antigens. Infect. Immun..

[38] Jumani R.S., Hasan M.M., Stebbins E.E., Donnelly L., Miller P., Klopfer C., Bessoff K., Teixeira J.E., Love M.S., McNamara C.W. (2019). A Suite of Phenotypic Assays to Ensure Pipeline Diversity When Prioritizing Drug-like *Cryptosporidium* Growth Inhibitors. Nat. Commun..

[39] O’Connor R.M., Wanyiri J.W., Cevallos A.M., Priest J.W., Ward H.D. (2007). *Cryptosporidium parvum* Glycoprotein Gp40 Localizes to the Sporozoite Surface by Association with Gp15. Mol. Biochem. Parasitol..

[40] Vinayak S., Pawlowic M.C., Sateriale A., Brooks C.F., Studstill C.J., Bar-Peled Y., Cipriano M.J., Striepen B. (2015). Genetic Modification of the Diarrhoeal Pathogen *Cryptosporidium parvum*. Nature.

[41] Chen X., Murawski A., Patel K., Crespi C.L., Balimane P.V. (2008). A Novel Design of Artificial Membrane for Improving the PAMPA Model. Pharm. Res..

[42] Daina A., Michielin O., Zoete V. (2017). SwissADME: A Free Web Tool to Evaluate Pharmacokinetics, Drug-Likeness and Medicinal Chemistry Friendliness of Small Molecules. Sci. Rep..

[43] Wang P.-H., Tu Y.-S., Tseng Y.J. (2019). PgpRules: A Decision Tree Based Prediction Server for P-Glycoprotein Substrates and Inhibitors. Bioinformatics.

[44] Bahar F.G., Ohura K., Ogihara T., Imai T. (2012). Species Difference of Esterase Expression and Hydrolase Activity in Plasma. J. Pharm. Sci..

[45] Funkhouser-Jones L.J., Ravindran S., Sibley L.D. (2020). Defining Stage-Specific Activity of Potent New Inhibitors of Cryptosporidium Parvum Growth in Vitro. mBio.

[46] Pane S., Putignani L. (2022). *Cryptosporidium*: Still Open Scenarios. Pathogens.

[47] Gorla S.K., McNair N.N., Yang G., Gao S., Hu M., Jala V.R., Haribabu B., Striepen B., Cuny G.D., Mead J.R. (2014). Validation of IMP Dehydrogenase Inhibitors in a Mouse Model of Cryptosporidiosis. Antimicrob. Agents Chemother..

[48] Castellanos-Gonzalez A., White A.C., Ojo K.K., Vidadala R.S.R., Zhang Z., Reid M.C., Fox A.M.W., Keyloun K.R., Rivas K., Irani A. (2013). A Novel Calcium-Dependent Protein Kinase Inhibitor as a Lead Compound for Treating Cryptosporidiosis. J. Infect. Dis..

[49] Guo F., Zhang H., Fritzler J.M., Rider S.D., Xiang L., McNair N.N., Mead J.R., Zhu G. (2014). Amelioration of *Cryptosporidium parvum* Infection in Vitro and in Vivo by Targeting Parasite Fatty Acyl-Coenzyme A Synthetases. J. Infect. Dis..

[50] Ndao M., Nath-Chowdhury M., Sajid M., Marcus V., Mashiyama S.T., Sakanari J., Chow E., Mackey Z., Land K.M., Jacobson M.P. (2013). A Cysteine Protease Inhibitor Rescues Mice from a Lethal *Cryptosporidium parvum* Infection. Antimicrob. Agents Chemother..

[51] Manjunatha U.H., Vinayak S., Zambriski J.A., Chao A.T., Sy T., Noble C.G., Bonamy G.M.C., Kondreddi R.R., Zou B., Gedeck P. (2017). A *Cryptosporidium* PI(4)K Inhibitor Is a Drug Candidate for Cryptosporidiosis. Nature.

[52] Jumani R.S., Bessoff K., Love M.S., Miller P., Stebbins E.E., Teixeira J.E., Campbell M.A., Meyers M.J., Zambriski J.A., Nunez V. (2018). A Novel Piperazine-Based Drug Lead for Cryptosporidiosis from the Medicines for Malaria Venture Open-Access Malaria Box. Antimicrob. Agents Chemother..

[53] Hulverson M.A., Vinayak S., Choi R., Schaefer D.A., Castellanos-Gonzalez A., Vidadala R.S.R., Brooks C.F., Herbert G.T., Betzer D.P., Whitman G.R. (2017). Bumped-Kinase Inhibitors for Cryptosporidiosis Therapy. J. Infect. Dis..

[54] Love M.S., Beasley F.C., Jumani R.S., Wright T.M., Chatterjee A.K., Huston C.D., Schultz P.G., McNamara C.W. (2017). A High-Throughput Phenotypic Screen Identifies Clofazimine as a Potential Treatment for Cryptosporidiosis. PLoS Negl. Trop. Dis..

[55] Huston C.D. (2021). The Clofazimine for Treatment of Cryptosporidiosis in HIV-Infected Adults (CRYPTOFAZ) and Lessons Learned for Anticryptosporidial Drug Development. Clin. Infect. Dis..

[56] Del Campo J., Heger T.J., Rodríguez-Martínez R., Worden A.Z., Richards T.A., Massana R., Keeling P.J. (2019). Assessing the Diversity and Distribution of Apicomplexans in Host and Free-Living Environments Using High-Throughput Amplicon Data and a Phylogenetically Informed Reference Framework. Front. Microbiol..

[57] John M., Krohn K., Flörke U., Aust H.-J., Draeger S., Schulz B. (1999). Biologically Active Secondary Metabolites from Fungi. 12. Oidiolactones A−F, Labdane Diterpene Derivatives Isolated from *Oidiodendron truncata*. J. Nat. Prod..

[58] Herath H.M.T.B., Herath W.H.M.W., Carvalho P., Khan S.I., Tekwani B.L., Duke S.O., Tomaso-Peterson M., Nanayakkara N.P.D. (2009). Biologically Active Tetranorditerpenoids from the Fungus *Sclerotinia homoeocarpa* Causal Agent of Dollar Spot in Turfgrass. J. Nat. Prod..

[59] Deng Z., Sheng F., Yang S.-Y., Liu Y., Zou L., Zhang L.-L. (2023). A Comprehensive Review on the Medicinal Usage of *Podocarpus* Species: Phytochemistry and Pharmacology. J. Ethnopharmacol..

[60] González-Coloma A., Reina M., Medinaveitia A., Guadaño A., Santana O., Martínez-Díaz R., Ruiz-Mesía L., Alva A., Grandez M., Díaz R. (2004). Structural Diversity and Defensive Properties of Norditerpenoid Alkaloids. J. Chem. Ecol..

[61] Barrero A.F., Quilez Del Moral J.F., Mar Herrador M., Rahman A.-U. (2003). Podolactones: A Group of Biologically Active Norditerpenoids. Studies in Natural Products Chemistry.

[62] Kubo I., Sutisna M., Kah-Siew T. (1991). Effects of Nagilactones on the Growth of Lettuce Seedlings. Phytochemistry.

[63] Manjunatha U.H., Lakshminarayana S.B., Jumani R.S., Chao A.T., Young J.M., Gable J.E., Knapp M., Hanna I., Galarneau J.-R., Cantwell J. (2024). *Cryptosporidium* PI(4)K Inhibitor EDI048 Is a Gut-Restricted Parasiticidal Agent to Treat Paediatric Enteric Cryptosporidiosis. Nat. Microbiol..

[64] Caldwell N., Peet C., Miller P., Colon B.L., Taylor M.G., Cocco M., Dawson A., Lukac I., Teixeira J.E., Robinson L. (2024). *Cryptosporidium* Lysyl-TRNA Synthetase Inhibitors Define the Interplay between Solubility and Permeability Required to Achieve Efficacy. Sci. Transl. Med..

[65] Arnold S.L.M., Choi R., Hulverson M.A., Schaefer D.A., Vinayak S., Vidadala R.S.R., McCloskey M.C., Whitman G.R., Huang W., Barrett L.K. (2017). Necessity of Bumped Kinase Inhibitor Gastrointestinal Exposure in Treating *Cryptosporidium* Infection. J. Infect. Dis..

[66] Amin M.L. (2013). P-glycoprotein Inhibition for Optimal Drug Delivery. Drug Target Insights.

[67] Arnold S.L.M., Choi R., Hulverson M.A., Whitman G.R., Mccloskey M.C., Dorr C.S., Vidadala R.S.R., Khatod M., Morada M., Barrett L.K. (2019). P-Glycoprotein-Mediated Efflux Reduces the in Vivo Efficacy of a Therapeutic Targeting the Gastrointestinal Parasite *Cryptosporidium*. J. Infect. Dis..

[68] Li D., Chen L., Li Y., Tian S., Sun H., Hou T. (2014). ADMET Evaluation in Drug Discovery. 13. Development of in Silico Prediction Models for P-Glycoprotein Substrates. Mol. Pharm..

[69] Desai P.V., Sawada G.A., Watson I.A., Raub T.J. (2013). Integration of in Silico and in Vitro Tools for Scaffold Optimization during Drug Discovery: Predicting P-Glycoprotein Efflux. Mol. Pharm..

[70] Hanessian S., Boyer N., Reddy G.J., Deschênes-Simard B. (2009). Total Synthesis of Oidiodendrolides and Related Norditerpene Dilactones from a Common Precursor: Metabolites CJ-14,445, LL-Z1271gamma, Oidiolactones A, B, C, and D, and Nagilactone F. Org. Lett..

[71] Zhang Y., Li X., Xu T. (2021). Total Synthesis of Bioactive Tetracyclic Norditerpene Dilactones. Org. Biomol. Chem..

[72] Barrero A.F., Arseniyadis S., Quílez del Moral J.F., Herrador M.M., Valdivia M., Jiménez D. (2002). First Synthesis of the Antifungal Oidiolactone C from Trans-Communic Acid: Cytotoxic and Antimicrobial Activity in Podolactone-Related Compounds. J. Org. Chem..

[73] Zhang Z., Miao L., Lv C., Sun H., Wei S., Wang B., Huang C., Jiao B. (2013). Wentilactone B Induces G2/M Phase Arrest and Apoptosis via the Ras/Raf/MAPK Signaling Pathway in Human Hepatoma SMMC-7721 Cells. Cell Death Dis..

[74] Zhang L.-L., Jiang X.-M., Huang M.-Y., Feng Z.-L., Chen X., Wang Y., Li H., Li A., Lin L.-G., Lu J.-J. (2019). Nagilactone E Suppresses TGF-Β1-Induced Epithelial-Mesenchymal Transition, Migration and Invasion in Non-Small Cell Lung Cancer Cells. Phytomedicine.

[75] Benatrehina P.A., Chen W.-L., Czarnecki A.A., Kurina S., Chai H.-B., Lantvit D.D., Ninh T.N., Zhang X., Soejarto D.D., Burdette J.E. (2019). Bioactivity-Guided Isolation of Totarane-Derived Diterpenes from *Podocarpus neriifolius* and Structure Revision of 3-Deoxy-2α-Hydroxynagilactone E. Nat. Products Bioprospect..

[76] Ichikawa K., Hirai H., Ishiguro M., Kambara T., Kato Y., Kim Y.J., Kojima Y., Matsunaga Y., Nishida H., Shiomi Y. (2001). Cytokine Production Inhibitors Produced by a Fungus, *Oidiodendron griseum*. J. Antibiot..

[77] Zhang L.-L., Guo J., Jiang X.-M., Chen X.-P., Wang Y.-T., Li A., Lin L.-G., Li H., Lu J.-J. (2020). Identification of Nagilactone E as a Protein Synthesis Inhibitor with Anticancer Activity. Acta Pharmacol. Sin..

[78] Devkota K.P., Ratnayake R., Colburn N.H., Wilson J.A., Henrich C.J., McMahon J.B., Beutler J.A. (2011). Inhibitors of the Oncogenic Transcription Factor AP-1 from *Podocarpus latifolius*. J. Nat. Prod..

[79] Bailly C. (2020). Anticancer Activities and Mechanism of Action of Nagilactones, a Group of Terpenoid Lactones Isolated from *Podocarpus* Species. Nat. Products Bioprospect..

