# Supplementary material for: Norditerpene Natural Products from Subterranean Fungi with Anti-Parasitic Activity
Source: Microorganisms. 2025 Nov 4;13(11):2527. doi: 10.3390/microorganisms13112527 (PMC12654037; doi:10.3390/microorganisms13112527)
Supplement: Supplementary file 1 [file microorganisms-13-02527-s001.zip › microorganisms-3911250-supplementary.pdf]

Supplementary Table S1: Activity of oidiolactones against *Plasmodium falciparum* isolates and HepG2 cells.

	EC_{50}		TC_{50}	
**Compound**	**Dd2 (μM)**	**3D7(μM)**	**HepG2 (μM)**	**SI**
1 oidiolactone A	2.263 ± 0.460	3.327 ± 0.640	> 25	>11
2 oidiolactone C	2.489 ± 0.198	2.777 ± 0.397	17.056 ± 0.821	7
3 oidiolactone D	> 5	> 5	> 25	>5
4 oidiolactone G	> 5	> 5	> 25	>5
5 oidiolactone H	n.t.	n.t.	n.t.	
6 oidiolactone I	> 4.985	3.651 ± 0.596	> 25	>5
7 oidiolactone B	0.983 ± 0.111	1.217 ± 0.419	10.334 ± 2.185	11
8 oidiolactone J	> 5	> 5	> 25	>5
9 oidiolactone K	> 5	> 5	> 25	>5
10 yukonin	n.t.	n.t.	n.t.	
11 oidiodendronic acid	> 5	> 5	> 25	>5
12 oidiolactone L	> 5	> 5	> 25	>5
13 oidiolactone E	> 5	> 5	> 25	>5
14 LL-Z1271β	> 5	> 5	> 25	>5

Data are presented as the mean +/- SD. SI: selectivity index=EC_{50} HepG2/ EC_{50} compound.
n.t.: not tested

Supplementary Table S2: Cytotoxicity of oidiolactones against confluent and sub-confluent host cells.

Compound	HCT-8 (confluent)	HCT-8 (sub-confluent)	HFF (confluent)	HuF (sub-confluent)[a]
1 oidiolactone A	>30	29.7 ± 1.8	>30	>30
2 oidiolactone C	>30	1.35 ± 0.2	>30	>30
3 oidiolactone D				>30
4 oidiolactone G				>30
5 oidiolactone H				>30
6 oidiolactone I	>30	4.8 ± 0.7	>30	>30
7 oidiolactone B	>30	2.1 ± 0.3	>30	21.4 ± .9
8 oidiolactone J	>30	>30	>30	>30
9 oidiolactone K				>30
10 yukonin	>30	2.5 ± 0.3	>30	n.t.
11 oidiodendronic acid	>30	19.7 ± 4.8	>30	>30
12 oidiolactone L				>30
13 oidiolactone E				n.t.
14 LL-Z1271β				>30

TC50 values are in µM. n.t.=not tested.[a]TC50 values for *Homo sapiens* HuF fibroblast cell line were previously reported in Rusman et al. 2020 [25].

Supplementary Table S3: Biochemical analysis of serum from infected and treated IFNγ -/- mice. Values represent the mean ± standard deviation (SD) of two mice per group.

Parameters	Sample ID	
	Oidiolactone A (mean ± SD)	Control(mean ± SD)
BUN (mg/dL)	21.50 ± 4.95	19.50 ± 0.71
Creat (mg/dL)	0.00 ± 0.00	0.00 ± 0.00
Ca (mg/dL)	10.55 ± 0.07	10.70 ± 0.28
Phos (mg/dL)	10.25 ± 0.35	11.00 ± 0.99
Mg (mg/dL)	2.75 ± 0.07	3.10 ± 0.14
TP (g/dL)	4.85 ± 0.21	5.05 ± 0.07
Alb (g/dL)	3.30 ± 0.14	3.40 ± 0.00
Glob (g/dL, calc.)	1.55 ± 0.07	1.65 ± 0.07
Na (mmol/L)	149.50 ± 0.71	151.50 ± 0.71
Cl (mmol/L)	112.50 ± 0.71	112.00 ± 0.00
K (mmol/L)	5.15 ± 0.07	5.60 ± 0.42
HCO3 (mmol/L)	14.85 ± 1.48	16.95 ± 0.21
Osmol (calc.)	310.00 ± 1.41	315.00 ± 2.83
An Gap (calc.)	27.00 ± 1.41	28.00 ± 0.00
T. Bili (mg/dL)	0.10 ± 0.00	0.15 ± 0.07
ALP (U/L)	182.00 ± 15.56	171.50 ± 14.85
GGT (U/L)	0.00 ± 0.00	0.00 ± 0.00
ALT (U/L)	37.50 ± 6.36	29.00 ± 9.90
AST (U/L)	229.50 ± 31.82	162.00 ± 69.30
CK (U/L)	1618.00 ± 376.18	1057.00 ± 339.41
Gluc (mg/dL)	280.00 ± 15.56	316.50 ± 30.41
Chol (mg/dL)	92.50 ± 7.78	106.00 ± 11.31
Amy (U/L)	456.00 ± 80.61	582.00 ± 63.64
LIH*	1.5 ± 0.71	0.0 ± 0.0

*LIH indicates the sum of lipemia, icterus, and hemolysis scores per mouse, presented as mean ± SD for each group.

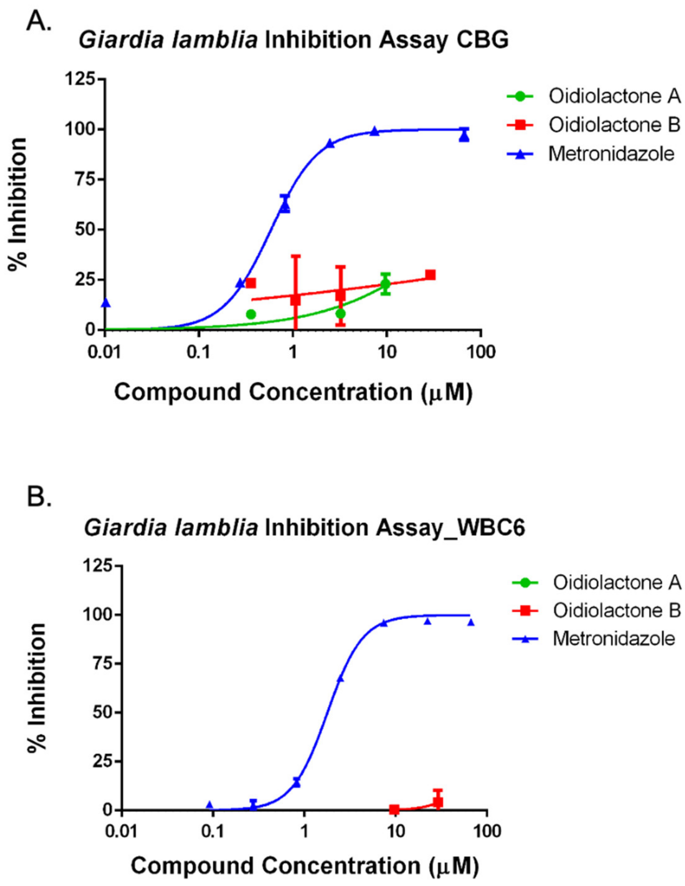

Supplementary Figure S1. Dose–response curves of oidiolactones A and B against CBG (A.) and WBC6 (B.) *G. lamblia* trophozoites.

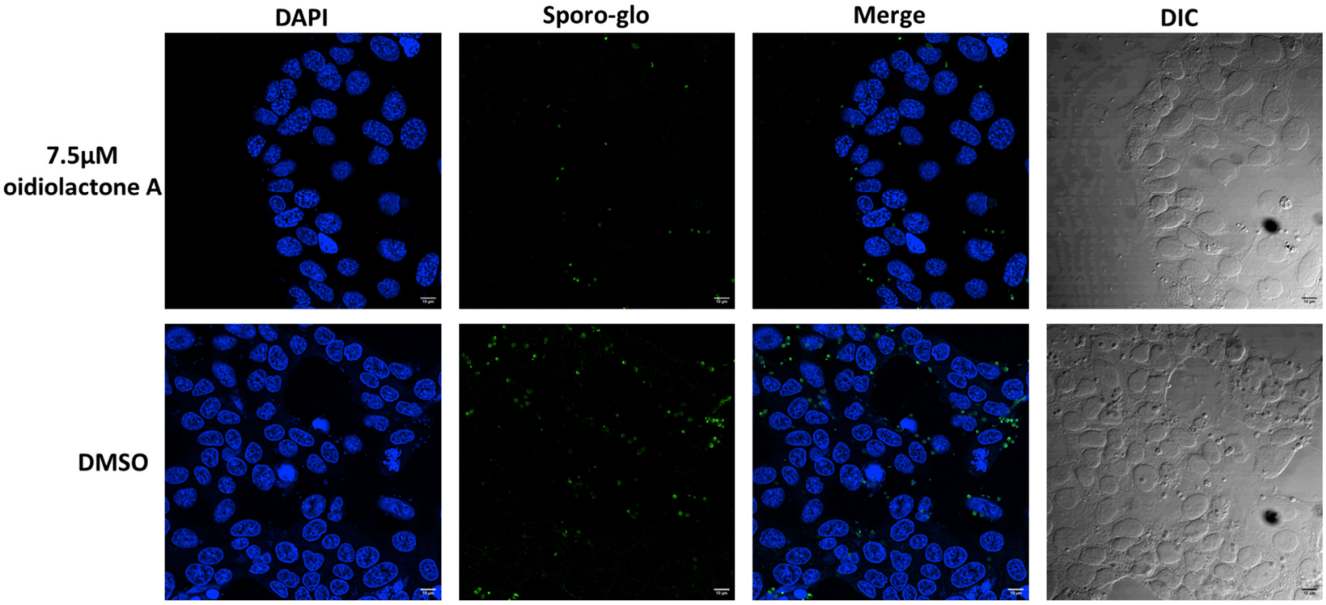

Supplementary Figure S2: ***C. parvum*** **intracellular stages treated with oidiolactone A**. HCT-8 cells were grown to confluency in 4-well chamber slides and then infected with *C. parvum* oocysts. At 4hpi, 7.5µM oidiolactone A or DMSO were added to the parasites. After 20 h of treatment, the samples were fixed, permeabilized, stained with Sporo-glo, and mounted with mounting media containing DAPI. The images were taken with a Nikon AX R microscope using the 60X oil objective. The scale bars are 10µm. Image processing was performed using Fiji v1.54p.

.

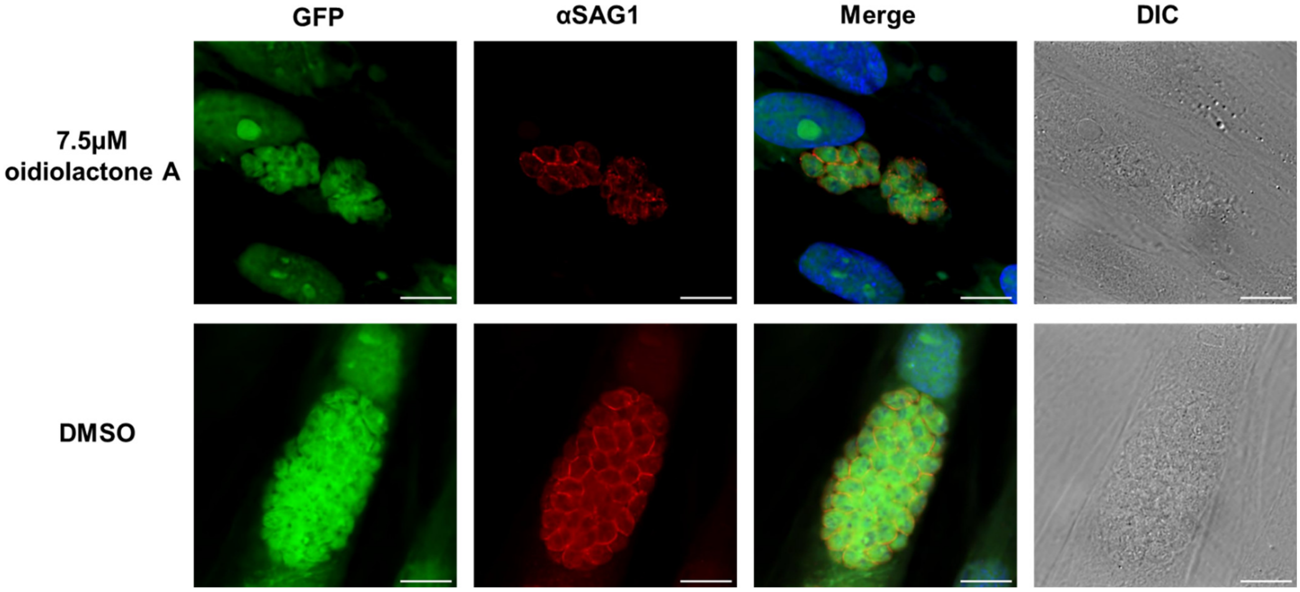

Supplementary Figure S3: ***T. gondii*** **intracellular stages treated with oidiolactone A**. HFF cells were grown to confluency in 4-well chamber slides and then infected with *T. gondii* tachyzoites expressing GFP and LUC. At 6hpi, 7.5µM oidiolactone A or DMSO were added to the parasites. After 24h of treatment, the samples were fixed, permeabilized, stained with anti-*T. gondii* SAG1 antibody, and visualized with Alexafluor 594 conjugated goat anti-rabbit IgG. Slides were mounted with mounting media containing DAPI to visualize nuclei (blue). The images were taken with a Nikon AX R microscope using the 60X oil objective. The scale bars are 10µm. Image processing was performed using NIS Elements GA3 (general analysis 3).

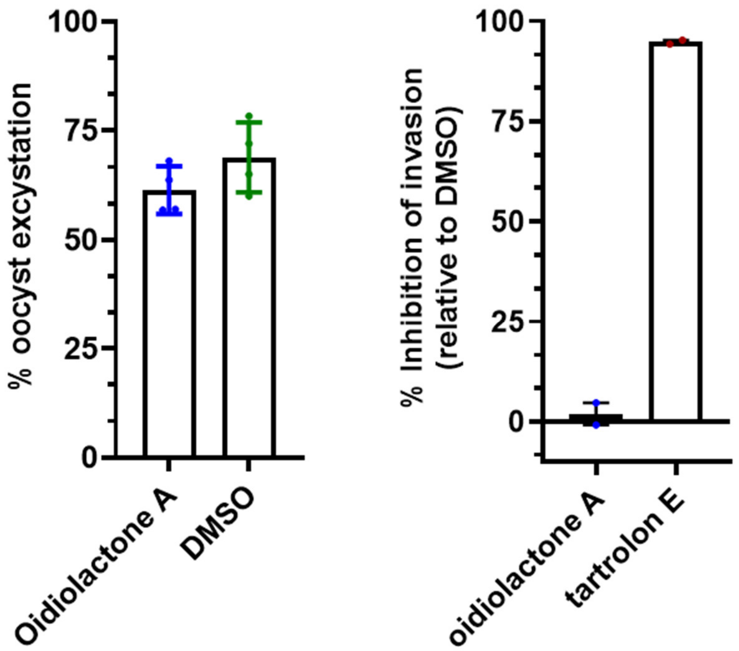

Supplementary Figure S4: Oidiolactone A has no effect on oocyst excystation or sporozoite invasion into host cells. (A) Oocysts were excysted in buffer containing 7.5µM odiolactone A or DMSO vehicle control. After a 1-hour incubation, the % of oocysts that had excysted was determined. Data were compiled from 4 biological replicates (n.s.=not significant). (B) WT *C. parvum* oocysts were added to HCT-8 cells in buffer containing 7.5 µM oidiolactone A, 100 nM tartrolon E, or DMSO and allowed to infect for 3 hours, at which point cells were washed, fixed, and stained with anti-gp40 and Hoechst. Slides were coded for unbiased counting of invaded parasites. Percent inhibition calculated by comparison to DMSO control. Data represent 2 biological replicates.
